# “Gas Station Heroin” in Plain Sight: Tianeptine Misuse, Detection Gaps, and the Role of Substance-Use Screening

**DOI:** 10.7759/cureus.117849

**Published:** 2026-10-13

**Authors:** Nelson J Tiburcio, Scarlett L Baker

**Affiliations:** 1 Substance Use and Addiction, The SASSI Institute, Inc., Springville, USA; 2 Training, Dissemination, and Planning, The SASSI Institute, Inc., Springville, USA

**Keywords:** gas station heroin, sassi, substance use screening, tianeptine, urine screening

## Abstract

Most often sold in the United States as a dietary supplement, nootropic, or research chemical, tianeptine has become better known by the troubling nickname “gas station heroin.” At high doses, it can produce opioid-like effects and lead to dependence, severe withdrawal, poisoning, and even death. This article traces how tianeptine entered the U.S. market and how it is promoted, resulting in the alarming present-day use trends. Attention is given to a large problem: tianeptine is typically excluded from conventional, immunoassay-based routine drug panels, necessitating targeted chromatographic-mass spectrometric toxicology panels for definitive identification. As a result, its use may go unnoticed in treatment programs, workplaces, and criminal justice settings. Although specialized toxicology tests can identify the drug, they are not widely used and are often costly. General substance-use screening instruments cannot establish tianeptine exposure alone; however, when used along with structured interviews, they can help identify broader substance-use risk by uncovering patterns of misuse, including the use of multiple substances and over-the-counter products. Asking direct questions and combining screening methods may help professionals recognize tianeptine use earlier so intervention can begin.

## Editorial

"Gas station heroin" is a slang nickname for tianeptine, an unregulated drug frequently sold online in the United States as well as over the counter at gas stations and convenience stores/smoke shops. It earned the nickname “gas station heroin” because, when taken in high doses, it binds to the brain's mu-opioid receptors, producing a euphoric high, sedation, and pain relief strikingly similar to heroin or morphine [1-3]. At the time of this publication, the Controlled Substances Act in the United States has not finalized regulatory action listing tianeptine as a Schedule I drug. However, in July of 2026, a proposed rule was entered into the Federal Register by the Drug Enforcement Administration (DEA) to place it in the Schedule I category, and it is awaiting adoption as a final rule [4]. The proposed rule states that 15 of the 50 United States have controlled or otherwise banned the sale of tianeptine, but these bans do not prohibit individuals from crossing state lines to buy it and have not limited the sale and availability of it online [4].

Additionally, the Food and Drug Administration (FDA) has not approved tianeptine for any medical use, and it does not meet the statutory definition of a dietary ingredient nor is it recognized as safe for use in food. Nevertheless, tianeptine is being marketed as a “research chemical,” a “nootropic” cognitive enhancer, or a dietary supplement [5-6]. In the United States, they are commonly advertised with these unproven claims and can be purchased as over-the-counter drugs. The Federal Trade Commission (FTC), as well as the FDA, have warned consumers and manufacturers about these possibly false supplement marketing scams for many years [7-9].

According to Operation Supplement Safety, the use of a "nootropic" was first documented in reference to substances purported to increase cognitive functions by Corneliu E. Giurgea in 1972 [10-12]. While researching a new compound, he found a spectrum of effects that did not align with any psychotropic drug category. This led to the concept of the term nootropic and his proposal of a new category. In his extensive report, Giurgea, among other findings, stated that nootropic drugs should enhance learning and memory, protect the brain against chemical or various physical injuries, and increase the efficacy of the tonic cortical control mechanisms [11]. Despite his findings, no clinical or globally accepted definition of a nootropic exists, and the majority of compounds described as nootropic do not presently align with Giurgea's characteristics [12].

Prescription drugs and dietary supplements both include nootropics marketed to enhance brain function, but while FDA approval establishes that a product has satisfied applicable standards for safety, efficacy, and quality for specified indications, it does not mean that all FDA-approved drugs have proven benefits [9]. Further, the FDA and FTC stated that some nootropic products, such as racetam drugs, vinpocetine, sulbutiamine, phenibut, and huperzine A, had not been approved for any medical purpose, were not demonstrated to be safe as a prescription-effective drug, and in the United States, were illegally marketed in violation of the Federal Food, Drug, and Cosmetic Act [7-8].

Here is what you need to know about its use, marketing, and the serious dangers associated with it.

Because it is not approved by the FDA for any medical use in the United States, manufacturers bypass federal regulations by marketing it as a "dietary supplement," "nootropic," or "research chemical." It is commonly packaged in colorful bottles or packets resembling energy shots under names like ZaZa, Tianna, Neptune’s Fix, Pegasus, and TD Red, among others [12-14].

Originally developed in France in the 1960s, in technical terms, tianeptine is an atypical tricyclic antidepressant [1,15]. While it is still approved/prescribed for depression in several countries, it has never been approved for medical use in the U.S. [16-18].

The dangers associated with tianeptine are numerous, highly toxic, and deadly. Tianeptine mimics opioids in high doses, so physical dependence is possible. Once they’ve built a tolerance, users require higher amounts to avoid getting sick or achieve the same effect [13]. These products lack regulation, so inconsistent dosing is more the rule than the exception [14]. Given the increasing number of negative events related to tianeptine, the FDA has issued public warnings urging consumers to avoid its purchase or use [13].

As indicated earlier, tianeptine is an antidepressant drug that is not FDA-approved. In the United States, even though it is not approved for use, it can be bought online or at small stores, smoke shops, and gas stations. It may be sold under varying creative and catchy marketing names, including ZaZa or Tianna Red. In many stores, tianeptine is labeled as a dietary supplement that stabilizes mood and enhances cognition [19]. Marketed as Stablon or Coaxil, in Europe, Asia, and Latin America, it is an atypical tricyclic drug used as an antidepressant [20]. The clinical effects of tianeptine abuse and withdrawal can mimic opioid toxicity and withdrawal. Some people take tianeptine with alcohol, opioids, or other drugs to heighten the effects. Tianeptine is sometimes misused to self-treat withdrawal symptoms from other substances, including alcohol. Many case studies on the recreational abuse of tianeptine have reported severe adverse effects and even death [21-24].

Routine 5-panel, 10-panel, and even 12-panel urine drug screen tests used by most parole/probation offices, clinics, and employers do not screen for tianeptine. Individuals who misuse tianeptine may combine it with other drugs and may be unaware of the drug’s dangers, and most urine assays do not screen for it [19]. Although tianeptine features a core structural similarity to classic tricyclic antidepressants, it exhibits a distinct pharmacokinetic profile that complicates traditional toxicological surveillance. Unlike traditional opioids or tricyclics, tianeptine escapes standard hospital urine drug screens (UDS) not because it is excreted unchanged, but due to its rapid and extensive hepatic biotransformation [25-26].

In a personal communication reflecting on his individual anecdotal experience, a recently retired Midwestern Community Corrections Director who served the criminal justice field for over 4 decades (10 years as a deputy warden and warden at a Midwest state correctional facility, and the subsequent 30 years with community corrections) gave us his permission to share the following: “Tianeptine use is often very difficult, if not impossible to detect.” He further related: “If the individual does not him or herself confess to the use of these gas station drugs or similar substances, there is no way for us to detect that they are using, and this is complicated further by the fact that they will test negative for the standard panels….” (in this case a twelve-panel urine toxicology screen). “Most of the time we will only catch them if they have violated some other judge-imposed term of their electronic monitoring sentence (ELMO), or if they test positive for other substances and then confess to also using the undetected substance/s in combination with the other drugs.” Many users combine tianeptine with other depressants (i.e., alcohol) or stimulants to alter or intensify its psychoactive effects. Mixing tianeptine with alcohol or sedative medications increases the risk of life-threatening toxicity. Despite the observational limitation of poison control call data (i.e., data is voluntarily reported, coding errors may have occurred, and limited clinical information), these calls showed that among 83 tianeptine exposures with noted coexposures, the most reported coexposures were to phenibut, ethanol, benzodiazepines, and opioids [20].

Indeed, the difficulty in detecting the drug in standard drug tests is one of the challenges in combating tianeptine misuse. Despite its structural similarities to traditional tricyclic antidepressants (TCAs), tianeptine does not cross-react with standard commercial immunoassay panels, and as a result, standard multi-panel screens routinely miss it, necessitating specialized testing methods [27]. Due to this, individuals who misuse tianeptine can still pass many standard drug tests. This further complicates monitoring efforts for use in at-risk populations.

One possibility to combat this issue presently would be to use non-biological screeners that inquire about such potential use and patterns of use. The Substance Abuse Subtle Screening Inventory (SASSI) is one instrument to consider to screen for drugs, alcohol, and/or opioid abuse to identify risk probability [28]. While a high probability result on the SASSI may indicate general substance-related risk or a possible disorder, it does not specifically identify tianeptine consumption. Determining the use of tianeptine requires a specially targeted toxicological screening and/or a direct clinical interview. However, pairing the SASSI results with a structured interview allows clinicians to bypass individual denial while gathering the detailed information needed to identify specific substances, like tianeptine, especially when reviewing endorsed client responses to some questions on the SASSI. Discussing an individual’s responses to certain questions may help form a larger view of their alcohol or drug use patterns and drug/s of choice, including over-the-counter ones, and open the door to clinical insight into polysubstance usage and the risks associated with such [29].

Tianeptine presents a growing concern because it is easy to obtain, produces opioid-like effects at high doses, and often goes undetected on routine drug screens. When products are sold over the counter, they may appear harmless to consumers. When lack of regulation is considered alongside the risks of dependence, withdrawal, polysubstance use, toxicity, and death, the need for better awareness and earlier identification becomes clear.

While no single screening method will solve this problem, targeted toxicology testing is needed when tianeptine use is suspected; however, testing alone may not identify every person who is at risk. A more practical approach is to combine specialty testing with a validated substance-use screener and a structured interview that asks directly about over-the-counter products and the use of alcohol, sedatives, opioids, or stimulants. Screening findings can then be used to guide further assessment rather than being treated as proof that a person has used tianeptine.

Reducing tianeptine-related harm will require cooperation among clinicians, toxicology laboratories, substance-use professionals, criminal justice agencies, public health officials, policymakers, retailers, and consumers. Professionals who work with at-risk populations should be familiar with the names and marketing of these products and should ask about them directly when use is suspected. Until routine testing and the final adoption of the current proposed rule to federally classify tianeptine as a Schedule I drug are in place, the availability of these products, informed screening, and careful clinical questioning remain among the most useful tools for identifying risk and connecting individuals with appropriate care.
